# Prognostic factors of survival in patients with lung cancer after low-dose computed tomography screening: a multivariate analysis of a lung cancer screening cohort in China

**DOI:** 10.1186/s12885-025-14036-9

**Published:** 2025-04-09

**Authors:** Jun Li, Hui-Lin Xu, Wei-Xi Li, Xiao-Yu Ma, Xiao-Hua Liu, Zuo-Feng Zhang

**Affiliations:** 1 Department of Non-Communicable Diseases Prevention and Control, Shanghai Minhang Center for Disease Control and Prevention, Shanghai, 201101 China; 2 Department of Epidemiology, Fielding School of Public Health, University of California, Los Angeles, CA 90095 USA

**Keywords:** Prognostic factors, Survival, Lung cancer, Low-dose CT, Early screening cohort, China

## Abstract

**Objective:**

This study aimed to evaluate the prognostic factors influencing the survival of patients with lung cancer identified from a lung cancer screening cohort in the community.

**Methods:**

A total of 25,310 eligible participants were enrolled in this population-based prospective cohort study, derived from a community lung cancer screening program started from 2013 to 2017. Survival analyses were conducted using the Kaplan–Meier method and the log-rank test. Cox proportional hazards regression models were utilized to identify prognostic factors, including demographic characteristics, risk factors, low-dose CT (LDCT) screening, and treatment information.

**Results:**

The screening cohort identified a total of 429 patients with lung cancer (276 men, 153 women) during the study period. The 1-year, 3-year, and 5-year survival rates were 74.4%, 59.4% and 54.5%, respectively. The prognostic factors discovered by the multivariate analysis include gender (male vs. female, HR: 2.96, 95% CI: 1.88–4.64), age (HR: 1.02, 95% CI: 1.00–1.05), personal monthly income (2000–3999 CNY vs. < 2000 CNY, HR: 0.70, 95% CI: 0.52–0.95), pathological type (small cell carcinoma vs. adenocarcinoma, HR: 2.55, 95% CI: 1.39–4.66), stage (IV vs. 0-I, HR: 5.21, 95% CI: 2.78–9.75; III vs. 0-I, HR: 3.81, 95% CI: 1.88–7.74), surgery (yes vs. no, HR: 0.36, 95% CI: 0.23–0.57), and KPS (HR: 0.98, 95% CI: 0.98–0.99) among lung cancer patients identified by the basic model. Furthermore, solid nodule (non-solid nodule vs. solid nodule, HR: 0.47, 95% CI: 0.23–0.96) and larger-sized nodule (HR: 1.02, 95% CI: 1.00–1.03) were associated with a worse prognosis for lung cancer in the LDCT screening model.

**Conclusion:**

Prognostic factors of patients with lung cancer detected by LDCT screening were identified, which could potentially guide clinicians in the decision-making process for lung cancer management and treatment. Further studies with larger sample sizes and more detailed follow-up data are warranted for prognostic prediction.

**Supplementary Information:**

The online version contains supplementary material available at 10.1186/s12885-025-14036-9.

## Introduction

Lung cancer is the leading cause of cancer-related deaths and has the highest incidence among cancers globally, with approximately 1.82 million deaths and 2.48 million new cases yearly according to GLOBOCAN 2022 [1]. The global health burden of lung cancer reported by GLOBOCAN showed that 59.6% of the new lung cancer cases and 61.9% of the deaths from lung cancer were reported in Asia [2]. The disease burden of lung cancer in China has also been heavy with the characteristics of the highest cancer-related mortality rate, high morbidity and the speed-up of incidence in females [3]. The prognosis of lung cancer is still poor [4], closely related to clinical stage and treatment [5]. The International Early Lung Cancer Program (I-ELCAP) indicated that the ten-year survival rate for clinical stage I lung cancer after surgical treatment could reach about 90%, whereas, most lung cancer was diagnosed at advanced stages [6]. Hence, early detection and early diagnosis of lung cancer make the disease amenable to curative treatment and reduce mortality [7]. Lung cancer screening (LCS) enables individuals to have earlier access to clinical treatments, which will largely improve both the prognosis as well as the quality of life. With the advantage of high sensitivity of small lung nodule detection, low-dose computed tomography (LDCT) has been widely used as a tool for LCS in recent years. It has been proven that LCS with LDCT can effectively reduce mortality from lung cancer and improve the early lung cancer detection rate in three large-scale randomized controlled trials including NELSON, NLST and LUSI [8–10]. A cohort study involving more than 1 million individuals undergoing baseline LDCT imaging for LCS also demonstrated a significant stage shift towards early-stage lung cancer [11].

The United States Preventive Services Task Force (USPSTF) recommends LCS with LDCT based on risk assessment using age and smoking history as the only factors, however, this approach may not be effective in LDCT screening for lung cancer in Taiwan [12]. Compared to the Western regions, Asia bears a greater health burden from lung cancer and has a lower prevalence of smoking among those diagnosed with the disease, implying Asian especially Asian women with lung cancers are more common in never-smokers. Not all countries in Asia have a government-sponsored LCS program, the recommendations and screening tools vary across countries [7].

In consideration of the high incidence, the high mortality rate and the poor prognosis of lung cancer, numerous oncological studies have focused on the prognostic factors for patients with lung cancer, especially with non-small cell lung cancer (NSCLC), including patients-related prognostic factors, histopathological prognostic factors and comprehensive molecular profiling [13], such as age, gender, comorbidities, tumor size, lymph node extension, TNM classification, histological type, inflammatory factors and genetic mutations [14–17]. These studies primarily focused on clinically diagnosed lung cancer cases rather than those detected through screening [18–20]. Notably, patients with lung cancer detected through screening are more likely to present with early-stage disease compared to those diagnosed through clinical means [21, 22]. In addition, disparities among ethnic groups, regions, aetiologies, subtypes of lung cancer, as well as population-level risk factors such as smoking behavior, will result in varying prognoses for individuals undergoing LDCT screening [7, 23]. Whether the prognostic factors for Chinese lung cancer patients undergoing LDCT scans for LCS differ from those of patients from clinically diagnosed or other regions remains an open question. This study aimed to evaluate the survival of patients with lung cancer detected in an early screening cohort in the community and to assess the prognostic factors of these patients, in order to provide in-depth insights into the better clinical decision-making, effective intervention and efficient management among lung cancer patients identified by the LDCT screening.

## Methods

### Study population

**Fig. 1:**
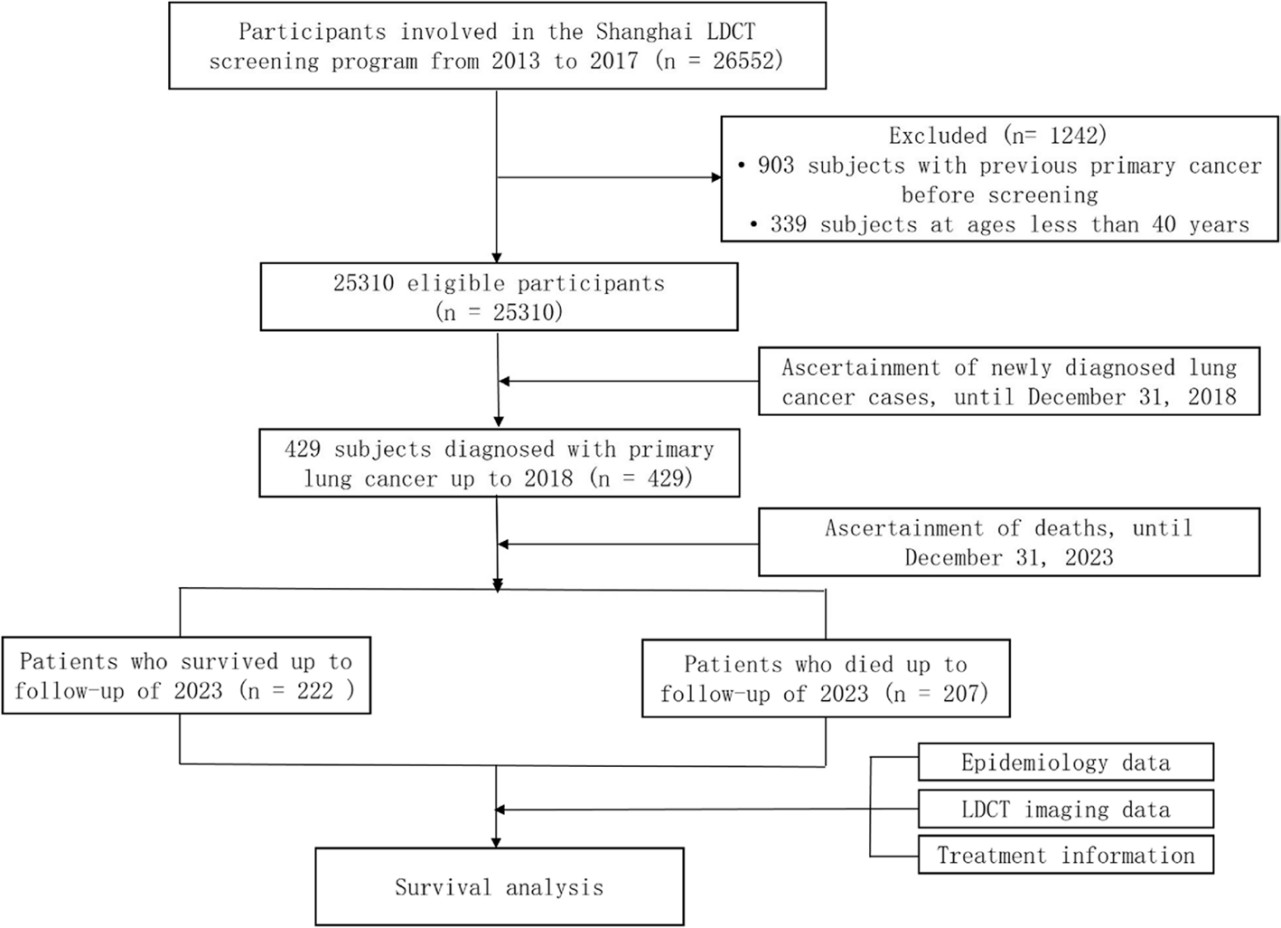
The flowchart for subject enrollment in the Shanghai Lung Cancer Screening program and the study design

This population-based prospective cohort study was based on a community lung cancer screening program implemented in Minhang district, southeastern Shanghai, China. During project implementation, all thirteen community health centers in Minhang district mobilized residents through various forms of mass media outreach, primarily consisted of flyers and brochures for residents, banners and posters in public places, and local radio and new media. A total of 26,552 community residential volunteers were enrolled in the study during the period from 2013 to 2017. The inclusion criterion for participants was an age of 40 years or older, and the exclusion criterion was a medical history of primary cancer. We excluded a total of 1,242 individuals who were not eligible for the study (903 individuals with prior primary cancer diagnoses before the beginning of the study and 339 individuals younger than 40 years old). Eventually, a total of 25,310 participants were included in the study (Fig. 1). All patients diagnosed with primary lung cancer have been followed up until date of death, or lost-to-follow-up, or December 31, 2023. The study was approved by Institutional Ethical Approval Committee of the Center for Disease Prevention and Control of Minhang district, Shanghai, China (No.: EC-P-2020–003).

### Data collection

#### The LDCT screening protocol

All participants who provided informed consent underwent LDCT screening at the Shanghai TCM-integrated Hospital. All scans were performed with a 64-slice multi-detector CT (Somatom Definition AS, Siemens Healthcare, Erlangen, Germany). The parameters of scanning were as follows: voltage control was 120 kV, current control was 30 mA, pitch was 1.25 mm. The data were reconstructed using filtered back projection, with a slice thickness of 1.5 mm and an increment of 1.5 mm. All the radiographic results were initially diagnosed by radiologists from the Shanghai TCM-integrated Hospital, and rechecked virtually by radiologists from Fudan University Shanghai Cancer Center. The nodules within the pulmonary parenchyma and bronchial lumen were described, with their sizes, positions, properties, and shapes recorded. The nodules were categorized as solid nodules, part-solid nodules, and non-solid nodules, the latter including ground-glass opacity (GGO) nodules. The nodule diameter was defined as an average of a long diameter and a vertical short diameter on the cross section of the largest nodule found in the lung window. In this study, LDCT evaluations were defined as positive if any noncalcified nodule ≥ 4 mm in diameter [24, 25]. Subsequently, in conjunction with clinical manifestations, patients with suspicious nodules were referred to specialized hospitals for further examination and diagnosis. Public health professionals from community health centers were responsible for the follow-up of these patients.

#### Data collection

The epidemiological data were collected through face-to-face interviews conducted by trained interviewers using a structured questionnaire (Supplementary file), included information on gender, age, education, personal monthly income, marital status, smoking status, and previous chronic obstructive pulmonary disease (COPD) or tuberculosis. Lung cancer patients’ electronic medical records have been reviewed and abstracted by a record linkage system with the Shanghai Cancer Registry. Clinical and treatment data, containing pathological type, clinical stage, surgery, chemotherapy, radiotherapy, and Karnofsky performance score (KPS), were obtained.

### Ascertainment of lung cancer cases and deaths

Newly diagnosed lung cancer cases up to December 31, 2018, and deaths among those patients with lung cancer up to December 31, 2023, were identified by a record linkage system with the Shanghai Cancer Registry and Shanghai Vital Statistics through the encrypted Resident Identity Card number.

### Statistical analysis

The primary endpoint of interest was overall survival (OS) of lung cancer, calculated from the date of lung cancer diagnosis to the date of death, or lost-to-follow-up, or the end of follow-up (December 31, 2023), whichever occurred first.

The categorical data were presented as percentages (%), and evaluated by Chi-square test. Continuous variables unsatisfying normal distribution were described as medians (interquartile range), and analyzed by Mann–Whitney U test. Survival curves were performed and compared using the Kaplan–Meier method and the log-rank test, respectively. Cox proportional hazards regression models were used to estimate adjusted hazard ratios (HRs) and the corresponding 95% confidence intervals (CIs), and to evaluate the prognostic factors of survival of patients with lung cancer after LDCT screening. We used scaled Schoenfeld residuals to evaluate the proportional hazards (PH) assumption for covariates. Two types of models were constructed: in the basic model, eleven factors were evaluated for survival including gender (male, female), age, education (primary school or below, high school, college or above), personal monthly income (< 2,000 ¥, 2,000–3999 ¥, ≥ 4000 ¥), marital status (married, single/divorced/widow/others), smoking status (never, ever), previous COPD or tuberculosis (yes, no), pathological type (adenocarcinoma, small cell carcinoma, squamous cell carcinoma, others), stage (0-I, II, III, IV, unknown), surgery (yes, no) and KPS; we further added nodule property (solid, part-solid, non-solid, uncertain) and nodule size (continuous) in the LDCT screening model.

All data analyses were performed in SPSS software package version 22.0 (IBM Corp., 2013), a two-sided *P*-value less than 0.05 was set as statistical significance.

## Results

Table 1 showed baseline demographic characteristics, risk factors, LDCT screening, and treatment information for both lung cancer patients alive and died groups. A total of 429 patients with LDCT diagnosed lung cancer were identified, including 276 (64.3%) men and 153 (35.7%) women, with median age of 66.4 years old. Over the 5-year follow-up period, 222 patients were alive and 207 patients died. The flowchart of subject enrollment was presented in Fig. 1. Patients who died during the follow-up period were predominantly male, older, less educated, lower income earners, smokers, and were treated with radiation and chemotherapy rather than surgical intervention. They also exhibited a lower detection rate and larger size of lung nodules, more solid nodules, more nonadenocarcinoma cases, more advanced lung cancer, and lower KPS (*P*-values < 0.05). There was no significant difference in marital status (*P*-value = 0.612) or previous COPD or tuberculosis (*P*-value = 0.309) with respect to survival status.

**Table 1:** Baseline demographic characteristics, risk factors, LDCT screening, and treatment information of lung cancer patients alive group and died group

Characteristics	Total (*N* = 429)	Lung Cancer Alive (*N* = 222)	Lung Cancer Died (*N* = 207)	^*2*^/Z	*P-*value
Gender, n (%)					
Female	153 (35.7)	128 (57.7)	25 (12.1)	96.992	0.000
Male	276 (64.3)	94 (42.3)	182 (87.9)		
Age (years), M (P25, P75)	66.4 (62.0, 70.9)	63.6 (60.1, 68.0)	68.7 (65.0, 74.7)	7.896	0.000
Education, n (%)					
Primary school or below	153 (35.7)	59 (26.6)	94 (45.4)	16.851	0.000
High school	264 (61.5)	155 (69.8)	109 (52.7)		
College or above	12 (2.8)	8 (3.6)	4 (1.9)		
Personal monthly income (CNY), n (%)				14.342	0.001
< 2000	232 (54.1)	101 (45.5)	131 (63.3)		
20,00–3,999	174 (40.6)	105 (47.3)	69 (33.3)		
≥ 4000	23 (5.4)	16 (7.2)	7 (3.4)		
Marital status, n (%)					
Married	408 (95.1)	210 (94.6)	198 (95.7)	0.257	0.612
Single/divorced/widow/others	21 (4.9)	12 (5.4)	9 (4.3)		
Smoking, n (%)				31.527	0.000
Never	254 (59.2)	160 (72.1)	94 (45.4)		
Ever	175 (40.8)	62 (27.9)	113 (54.6)		
Previous COPD or tuberculosis, n (%)				1.035	0.309
No	405 (94.4)	212 (95.5)	193 (93.2)		
Yes	24 (5.6)	10 (4.5)	14 (6.8)		
Nodule, n (%)				16.489	0.000
No	157 (36.6)	61 (27.5)	96 (46.4)		
Yes	272 (63.4)	161 (72.5)	111 (53.6)		
Nodule property, n (%)				61.524	0.000
Solid nodule	109 (40.1)	45 (28.0)	64 (57.7)		
Part-solid nodule	20 (7.4)	16 (9.9)	4 (3.6)		
Non-solid nodule	93 (34.2)	82 (50.9)	11 (9.9)		
Uncertain nodule	50 (18.4)	18 (11.2)	32 (28.8)		
Nodule size (mm), M (P25, P75)	15.0 (10.0, 22.0)	13.0 (8.3, 16.0)	18.0 (12.0, 27.0)	4.382	0.000
Pathological type, n (%)					
Adenocarcinoma	214 (49.9)	168 (75.7)	46 (22.2)	124.444	0.000
Small cell carcinoma	19 (4.4)	2 (0.9)	17 (8.2)		
Squamous cell carcinoma	23 (5.4)	5 (2.3)	18 (8.7)		
Others^a^	173 (40.3)	47 (21.2)	126 (60.9)		
Stage, n (%)				104.058	0.000
0-I	123 (28.7)	107 (48.2)	16 (7.7)		
II	13 (3.0)	7 (3.2)	6 (2.9)		
III	25 (5.8)	5 (2.3)	20 (9.7)		
IV	52 (12.1)	8 (3.6)	44 (21.3)		
Unknown	216 (50.3)	95 (42.8)	121 (58.5)		
Surgery, n (%)				191.936	0.000
No	196 (45.7)	30 (13.5)	166 (80.2)		
Yes	233 (54.3)	192 (86.5)	41 (19.8)		
Chemotherapy, n (%)				12.758	0.000
No	349 (81.4)	195 (87.8)	154 (74.4)		
Yes	80 (18.6)	27 (12.2)	53 (25.6)		
Radiotherapy, n (%)				14.657	0.000
No	403 (93.9)	218 (98.2)	185 (89.4)		
Yes	26 (6.1)	4 (1.8)	22 (10.6)		
KPS^b^, M (P25, P75)	80 (47.5, 90)	90 (80, 90)	45 (0, 80)	11.708	0.000

^a^Others: including 11 cases of non-small cell lung carcinoma and 162 cases of pathologically unclassified lung cancer

^b^KPS: Karnofsky performance score

**Fig. 2:**
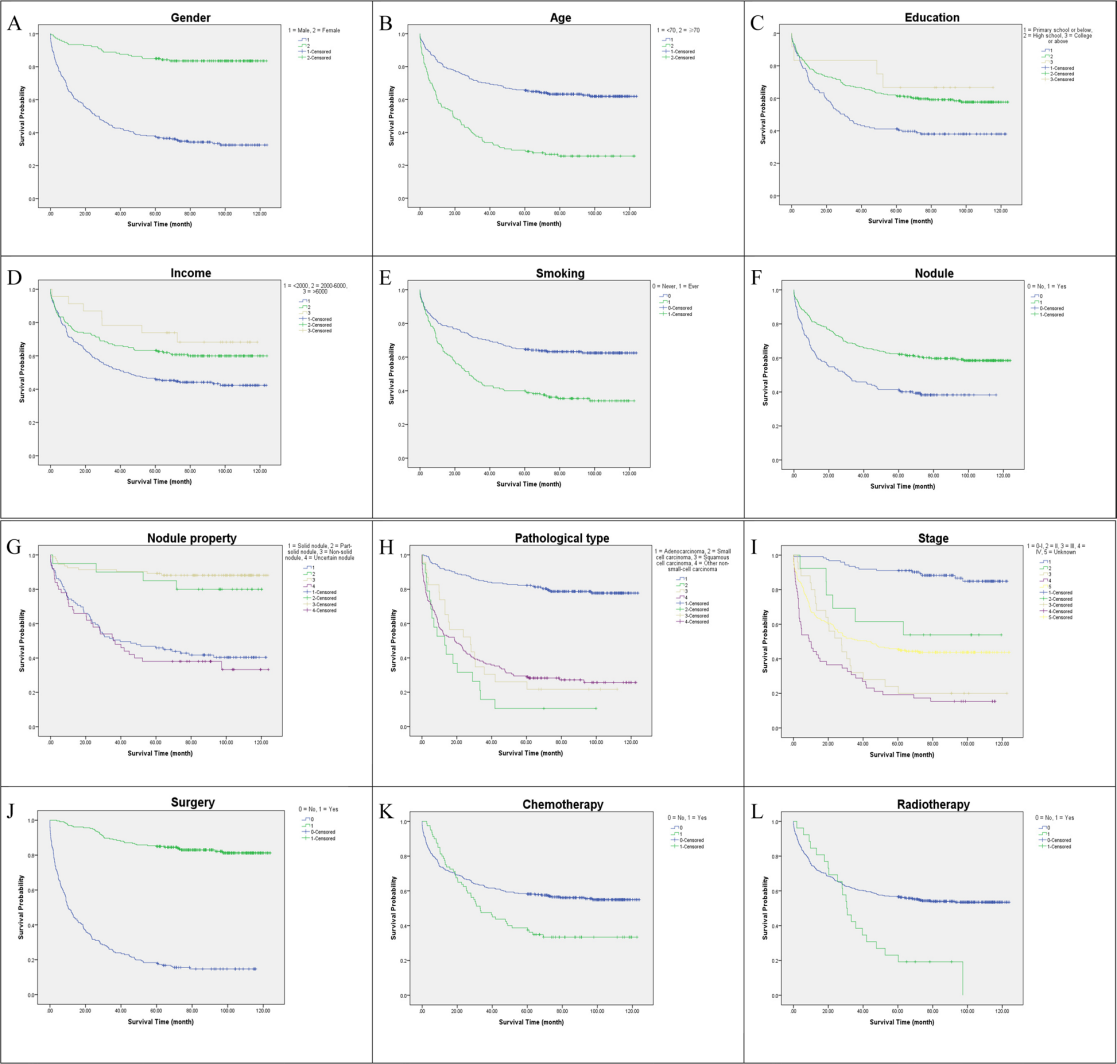
Survival curves for patients with lung cancer after LDCT screening, grouped by characteristics of (**a**) sex, (**b**) age, (**c**) education, (**d**) income, (**e**) smoking, (**f**) nodule, (**g**) nodule property, (**h**) pathological type, (**i**) stage, (**j**) surgery, (**k**) chemotherapy, (**l**) radiotherapy

Table 2 presented the univariate analysis of factors affecting lung cancer survival after LDCT screening. The mean survival time in this study, calculated from the date of diagnosis, was 72.7 months (95% CI: 67.6–77.9 months). The 1-year, 3-year, and 5-year survival rates were 74.4%, 59.4% and 54.5%, respectively. As shown in Table 2 and Fig. 2, the factors including gender, age, education, personal monthly income, smoking, nodule, nodule property, pathological type, stage, surgery, chemotherapy and radiotherapy, were associated with the mean survival time of lung cancer patients. To be specific, the mean survival time of females (107.6 months) was longer than that of males (53.2 months), additionally, patients who were younger (85.3 months), well-educated (85.5 months), relatively high-income (91.1 months), non-smokers (84.8 months), detected with non-solid nodule (111.2 months), diagnosed with adenocarcinoma (103.1 months), in the early stages of lung cancer (111.8 months), treated with surgery (108.0 months), tended to have a comparatively longer mean survival time (*P*-values < 0.05) and a higher survival rate. Compared to lung cancer patients who did not undergo radiotherapy or chemotherapy, their 1-year survival rate was higher, however, their 3-year and 5-year survival rates were lower.

**Table 2:** Univariate analysis of prognostic factors for patients with lung cancer after LDCT screening

Characteristics	n (%)	Survival rate (%)			MST^a^(month)	95%CI^b^ (month)	*P*-value
		1-year	3-year	5-year			
Total	429	74.4	59.4	54.5	72.7	67.6–77.9	
Gender, n (%)							
Female	153 (35.7)	93.5	88.9	85.0	107.6	101.7–113.5	0.000
Male	276 (64.3)	63.8	43.1	37.7	53.2	47.0–59.4	
Age (years), n (%)							
< 70	299 (69.7)	81.9	70.2	65.6	85.3	79.4–91.1	0.000
≥ 70	130 (30.3)	56.9	34.6	29.2	43.6	35.1–52.0	
Education, n (%)							
Primary school or below	153 (35.7)	68.0	44.4	41.2	57.3	48.9–65.7	0.000
High school	264 (61.5)	77.7	67.0	61.7	80.5	74.1–87.0	
College or above	12 (2.8)	83.3	66.7	66.7	85.5	60.2–110.8	
Personal monthly income (CNY), n (%)							0.001
< 2000	232 (54.1)	70.7	52.2	46.1	63.9	56.9–70.8	
20,00–3,999	174 (40.6)	77.0	66.7	63.2	81.4	73.5–89.4	
≥ 4000	23 (5.4)	91.3	78.3	73.9	91.1	73.6–108.5	
Marital status, n (%)							
Married	408 (95.1)	74.5	59.3	54.4	72.5	67.3–77.8	0.749
Single/divorced/widow/others	21 (4.9)	71.4	61.9	57.1	71.5	49.3–93.8	
Smoking, n (%)							0.000
Never	254 (59.2)	79.5	70.5	64.6	84.8	78.4–91.2	
Ever	175 (40.8)	67.4	43.4	40.0	54.7	47.0–62.4	
Previous COPD or tuberculosis, n (%)							
No	405 (94.4)	74.8	60.2	55.3	73.5	68.2–78.8	0.229
Yes	24 (5.6)	66.7	45.8	41.7	56.5	36.0–76.9	
Nodule, n (%)							0.000
No	157 (36.6)	63.1	45.9	41.4	53.9	46.0–61.9	
Yes	272 (63.4)	80.9	67.3	62.1	81.8	75.6–88.0	
Nodule property, n (%)							0.000
Solid nodule	109 (40.1)	73.4	50.5	45.9	62.6	52.8–72.5	
Part-solid nodule	20 (7.4)	95.0	90.0	85.0	103.7	88.3–119.0	
Non-solid nodule	93 (34.2)	92.5	91.4	89.2	111.2	104.2–118.3	
Uncertain nodule	50 (18.4)	70.0	50.0	38.0	57.2	42.8–71.6	
Pathological type, n (%)							0.000
Adenocarcinoma	214 (49.9)	92.1	84.6	82.2	103.1	97.5–108.6	
Small cell carcinoma	19 (4.4)	52.6	15.8	10.5	23.2	10.2–36.2	
Squamous cell carcinoma	23 (5.4)	73.9	30.4	26.1	40.2	23.8–56.6	
Others^c^	173 (40.3)	54.9	37.0	28.9	43.9	36.5–51.4	
Stage, n (%)							0.000
0-I	123 (28.7)	99.2	92.7	91.1	111.8	106.7–117.0	
II	13 (3.0)	92.3	61.5	61.5	76.8	50.9–102.7	
III	25 (5.8)	80.0	32.0	24.0	42.7	26.1–59.3	
IV	52 (12.1)	44.2	28.8	19.2	30.5	19.5–41.6	
Unknown	216 (50.3)	66.2	50.9	45.4	62.9	55.5–70.3	
Surgery, n (%)							0.000
No	196 (45.7)	47.4	24.5	18.4	29.6	24.1–35.1	
Yes	233 (54.3)	97.0	88.8	85.0	108.0	103.4–112.5	
Chemotherapy, n (%)							0.006
No	349 (81.4)	73.1	62.2	58.2	76.2	70.4–82.0	
Yes	80 (18.6)	80.0	47.5	38.8	57.2	46.6–67.9	
Radiotherapy, n (%)							0.003
No	403 (93.9)	73.7	60.8	56.6	74.6	69.2–79.9	
Yes	26 (6.1)	84.6	38.5	23.1	41.1	29.1–53.1	

^a^MST: mean survival time, no use of median survival time was for its deficiency of some variables

^b^CI: confidence interval

^c^Others: including 11 cases of non-small cell lung carcinoma and 162 cases of pathologically unclassified lung cancer

Table 3 demonstrated the results from multivariate Cox regression analysis of prognostic factors for patients with lung cancer in a community early screening cohort using two models involving different variables. We employed scaled Schoenfeld residuals to assess the PH assumption. Among all predictors, the PH assumption was met for gender (*P-value* = 0.910), age (*P-values* = 0.141), education (*P-value* = 0.076), marital status (*P-value* = 0.537), personal monthly income (*P-value* = 0.627), smoking (*P-value* = 0.107), previous COPD or tuberculosis (*P-value* = 0.505), surgery (*P-value* = 0.063), pathological type (*P-value* = 0.445), stage (*P-value* = 0.174), nodule (*P-value* = 0.103), and nodule property (*P-value* = 0.253). However, neither radiotherapy nor chemotherapy met the PH assumption; no suitable transformation was identified for these variables, and incorporating these two time-dependent covariates did not improve the model's goodness of fit. Consequently, they were treated as unadjusted confounding factors. The basic model integrated baseline demographic characteristics such as gender, age, education, personal monthly income, and marital status, along with risk factors including smoking status, previous COPD or tuberculosis, and treatment information such as pathological type, stage, surgery, and KPS. We further added nodule property and nodule size to the basic model as the LDCT screening model.

**Table 3:** Multivariate Cox regression analysis of prognostic factors for patients with lung cancer after LDCT screening

Basic Model^1^				LDCT Screening Model^2c^			
Factors	*P*-value	HR^a^	95% CI^b^	Factors	*P*-value	HR^a^	95% CI^b^
Gender				Gender			
Female		1	(Ref)	Female		1	(Ref)
Male	0.000	2.955	1.884–4.636	Male	0.005	2.308	1.280–4.162
Age (years)	0.042	1.024	1.001–1.047	Personal monthly income (CNY)			
				< 2000		1	(Ref)
Personal monthly income (CNY)				20,00–3,999	0.021	0.595	0.382–0.926
< 2000		1	(Ref)	≥ 4000	0.496	0.688	0.234–2.018
20,00–3,999	0.024	0.703	0.518–0.954	Pathological type			
≥ 4000	0.490	0.757	0.344–1.688	Adenocarcinoma		1	(Ref)
Pathological type				Small cell carcinoma	0.006	4.157	1.494–11.569
Adenocarcinoma		1	(Ref)	Squamous cell carcinoma	0.065	2.386	0.946–6.016
Small cell carcinoma	0.002	2.545	1.389–4.661	Others^d^	0.279	1.355	0.782–2.349
Squamous cell carcinoma	0.388	1.301	0.716–2.366	Stage			
Others^d^	0.075	1.441	0.964–2.155	0-I		1	(Ref)
Stage				II	0.031	3.818	1.133–12.868
0-I		1	(Ref)	III	0.017	3.255	1.239–8.551
II	0.230	1.806	0.688–4.741	IV	0.011	3.144	1.297–7.621
III	0.000	3.810	1.877–7.735	Unknown	0.093	1.960	0.895–4.296
IV	0.000	5.206	2.779–9.751	Surgery			
Unknown	0.002	2.426	1.386–4.244	No		1	(Ref)
Surgery				Yes	0.000	0.308	0.165–0.575
No		1	(Ref)	KPS^e^	0.000	0.981	0.974–0.989
Yes	0.000	0.364	0.231–0.574	Nodule property			
KPS^e^	0.000	0.982	0.978–0.986	Solid nodule		1	(Ref)
				Part-solid nodule	0.974	1.018	0.348–2.983
				Non-solid nodule	0.038	0.470	0.231–0.959
				Uncertain nodule	0.066	0.649	0.409–1.029
				Nodule size (mm)	0.030	1.015	1.001–1.028

^a^HR: hazard ratio

^b^CI: confidence interval

^c^LDCT Screening Model: 258 participants with description of nodule size were involved in the LDCT screening model

^d^Others: including 11 cases of non-small cell lung carcinoma and 162 cases of pathologically unclassified lung cancer

^e^KPS: Karnofsky performance score

^1^Involved gender (male, female), age (continuous), education (primary school or below, high school or above), personal monthly income (< 2,000¥, 2,000–3999¥, ≧4000¥), marital status (married, single/divorced/widow/others), smoking status (never, ever), previous COPD or tuberculosis (yes, no), pathological type (adenocarcinoma, small cell carcinoma, squamous cell carcinoma, others), stage (0-I, II, III, IV, unknown), surgery (yes, no), and KPS (continuous)

^2^Involved gender (male, female), age (continuous), education (primary school or below, high school or above), personal monthly income (< 2,000¥, 2,000–3999¥, ≧4000¥), marital status (married, single/divorced/widow/others), smoking status (never, ever), previous COPD or tuberculosis(yes, no), pathological type (adenocarcinoma, small cell carcinoma, squamous cell carcinoma, others), stage (0-I, II, III, IV, unknown), surgery (yes, no), KPS (continuous), nodule property (solid, part-solid, non-solid, uncertain), and nodule size (continuous)

In the basic model, gender (male vs. female, HR: 2.955, 95% CI: 1.884–4.636), age (HR: 1.024, 95% CI: 1.001–1.047), personal monthly income (2000–3999 CNY vs. < 2000 CNY, HR: 0.703, 95%CI: 0.518–0.954), pathological type (small cell carcinoma vs. adenocarcinoma, HR: 2.545, 95% CI: 1.389–4.661), stage (IV vs. 0-I, HR: 5.206, 95% CI: 2.779–9.751; III vs. 0-I, HR: 3.810, 95% CI: 1.877–7.735), surgical operation (yes vs. no, HR: 0.364, 95% CI: 0.231–0.574), and KPS (HR: 0.982, 95% CI: 0.978–0.986) were identified as independent factors affecting the survival of patients with lung cancer. With regard to the LDCT screening model, nodule property (non-solid nodule vs. solid nodule, HR: 0.470, 95% CI: 0.231–0.959) and nodule size (HR: 1.015, 95% CI: 1.001–1.028) were newly identified prognostic factors which exerted strong effects on the survival of patients. That is to say, in the basic model, older, male, and lower income were risk factors for the survival of patients, while adenocarcinoma, early stage, surgical intervention, and a high KPS were identified as protective factors. Furthermore, both solid nodule and larger-sized nodule were associated with a worse prognosis for lung cancer in the LDCT screening model.

## Discussion

Among patients with lung cancer detected by LDCT in a population-based prospective lung cancer screening program in Shanghai, China, we discovered that gender, age, income, pathological type, stage, surgery, and KPS were independent prognostic factors affecting survival in patients with screen-detected lung cancer. Specifically, our study revealed that older, male, and lower income were risk factors for the survival of patients, while adenocarcinoma, early stage, surgical intervention, and a high KPS were identified as protective factors. Particularly, the characteristics of pulmonary nodules detected by LDCT indicated that both solid nodule and larger-sized nodule were associated with a worse prognosis for lung cancer.

There were three demographic characteristics identified as independent prognostic factors in our study. Older patients had a worse prognosis (HR: 1.024, 95% CI: 1.001–1.047), which was consistent with previous studies [14, 18, 26, 27]. They may have more comorbidities and more treatment-related adverse events [28, 29]. A study reported that the underlying pathogenetic mechanisms of aging and lung cancer included microbiota disorders, immune mircoenviroment, epigenetics, metabolic disorder, genetics, endocrine disorder, proteostasis and cellular senescence [29]. Regarding gender, we found that male patients with lung cancer had a risk of death more than twice that of female patients (HR: 2.955, 95% CI: 1.884–4.636). Zhang et al*.* found a poorer survival outcome of NSCLC with ipsilateral pulmonary metastasis (IPM) in male patients compared to female patients (HR 1.845, 95% CI: 1.558–2.184) [14], Abrão et al*.* observed an increased risk of 52% for female patients with stage I non-small-cell lung cancer (NSCLC) (adjusted HR: 1.52, 95% CI: 1.17–1.98) [15]. Income is an important social determinant of health (SDOH), affecting the reduced ability to access cancer screening and treatment [30–32]. Previous studies showed that low income was associated with failure to undergo surgical resection in patients with stage I NSCLC [33]. In our study, relatively higher income in the two multivariate models demonstrated a protective effect on the survival of patients with screen-detected lung cancer.

With respect to pathological type, compared with adenocarcinoma, squamous cell carcinoma showed a borderline increased risk of death in the LDCT screening model (HR: 2.386, 95% CI: 0.946–6.016), while the risk of death related to small cell carcinoma was significantly higher in both models (HR: 2.545, 95% CI: 1.389–4.661; HR: 4.157, 95% CI: 1.494–11.569). It is well-known that small-cell lung cancer (SCLC) proliferates more rapidly and has a high propensity to metastasise. In a large study involved 359,873 patients who were diagnosed with a first primary lung cancer, the survival of 465 patients with resected SCLC was lower than patients with resected NSCLC (5-year survival 31% and 45%, respectively) and the HR compared with NSCLC was 1.47 [34]. A study based on the National Lung Screening Trial (NLST) demonstrated that mortality risk ratios (RRs) were 0.75 for adenocarcinoma, 1.23 for squamous cell carcinoma, and 0.90 for small cell carcinoma, in other words, the risk of dying from lung adenocarcinoma was relatively lower [35]. Other studies found that the stage-specific 5-year overall survival rate of adenocarcinoma cell carcinoma of lung cancer was higher than that of squamous cell carcinoma at the same clinical stage, which may be related to more specific treatment options for adenocarcinoma cell carcinoma after recurrence and metastasis [36, 37].

Clinical and pathological stage of lung cancer is vital to prognostic evaluation and treatment strategy development. Patients diagnosed with stage III/IV lung cancer had an increased risk of death than those diagnosed with stage 0-I lung cancer, consistent with those of previous studies revealing strong correlation between advanced lung cancer stage and the risk of death [14, 38, 39]. Regarding the therapeutic factor, surgery emerged as a significant prognostic indicator for survival in our multivariate analysis. When compared with lung cancer patients who did not undergo radiotherapy or chemotherapy, the mean survival time for those who received these treatments was shorter, and their 3-year and 5-year survival rates were lower. We observed that surgical operation reduced the risk of death by 64% and 69% in the basic model and in the LDCT screening model, respectively. Generally, the standard of care for early stage lung cancer is surgery [40, 41], chemotherapy is indicated for those with advanced stage lung cancer or used as an adjuvant therapy for patients who are deemed medically inoperable owing to surgery rejection and comorbidities [42–46]. Berry et al*.* found the 5-year all-cause survival of patients with clinical stage I NSCLC after no therapy (12.7%) was significantly worse than that of patients who underwent surgery (64.9%) [47]. KPS is an important scale for clinical assessment of the health status of malignant patients. A higher score is associated with better health status of patients and greater tolerance for the side effects caused by treatment. We found that a higher KPS played a protective role in improving the survival rate, in agreement with a study on patients with EGFR-mutated NSCLC, which indicated that KPS ≥ 70 was an independent prognostic factor of longer overall survival (HR: 0.391, 95%CI: 0.249–0.614) [48]. Determination of lung nodule malignancy is pivotal for the early diagnosis, intervention, and therapy of lung cancer. Previous studies have demonstrated the favorable prognostic impact of the presence of a ground-glass opacity (GGO) component in early-stage NSCLC [49, 50], corresponding to the pathological finding of less tumour invasion related to progress, similarly, we confirmed that the presence of the non-solid nodule was identified as an independently significant protective factor when compared to the solid nodule, which reduced the risk of death by 53%. Our study revealed that nodule size (HR: 1.015, 95% CI: 1.001–1.028) was significantly prognostic factor for survival, as the size of a nodule increases, so does the probability of death for screen-detected nodules. As we know, nodule size remains the primary predictor to evaluate the likelihood of nodule malignancy and to determine nodule management [51–53], the probability of malignancy for nodules is associated with an increase in nodule size [54]. It was reported that the prevalence of malignancy in nodules measuring below 5 mm ranged from 0 to 1% [55], and the prevalence of lung cancer among patients with nodules measuring 4–6 mm was also very low in the NLST [56]. Clinically, these evidences provide support for monitoring, assessment, and clinical decision-making regarding nodules. Based on the size of nodules from an initial screening scan, combined with their characteristics, intervals for reassessment scans and risk evaluations can be established. For patients who are candidates for curative surgery, surgical treatment may be adopted; alternatively, other local treatments such as targeted radiotherapy and radiofrequency ablation should be considered.

There are still several limitations in this study. Firstly, the risk factors of the participants were self-reported, which might lead to misclassification due to reporting bias, attenuating the true effect towards null. Secondly, risk factors included in the analysis were only measured at baseline and we could not evaluate the changes that occurred during follow-up. Thirdly, the relatively higher 5-year survival rate among patients with screen-detected lung cancer could be partly attributed to lead time bias. Although using death as the endpoint in the survival analysis could reduce lead time bias to some extent, it remained necessary to further mitigate this bias through various methods, such as extending follow-up periods or adopting randomized controlled trials. Fourthly, there were still other potential confounders that we were unable to control, such as smoking amounts, radiotherapy, chemotherapy, comorbidities, occupational exposure, as well as the stability of nodules, metastatic invasion, tumor size, mutations or gene fusions, so that we might be prone to overestimate the observed associations. Fifthly, volunteers undergoing LCS tended to have better knowledge and healthier behaviors, which might indicate different risk profiles from the general population, our study may be affected by potential selection bias. Finally, the sample size was comparatively small and the follow-up period was not long enough, which could affect the robustness of the statistics.

It is reported that only about 15% of lung cancers are at the stage I when diagnosed in routine clinical practice [21], after implementation of lung cancer screening by LDCT, a migration to earlier stage will occur [57], thus resulting in a shift in lung cancer management and treatment approaches of this growing patient population. Despite the controversy surrounding the target population and the issue of overdiagnosis among female never-smokers [58–61], where the incidence of early-stage lung cancer increased while the incidence of late-stage lung cancer remained stable during the same period, our study aimed to identify prognostic factors for the survival of patients with lung cancer undergoing LDCT scans for LCS. In summary, this prognostic study has gained deeper insights into the prognostic factors affecting the survival of patients with lung cancer detected through LDCT screening, considering comprehensive data consisted of demographic characteristics, risk factors, LDCT screening, and treatment information in Chinese real-world population. Attention must be given to vulnerable populations, especially the elderly and males. LDCT scans should be continuously employed for LCS in populations at high risk for lung cancer development, to diagnose the disease at an early stage when it is more amenable to curative treatments, such as surgery. It is necessary for medical professionals to assess the change or stability of nodules and to dynamically monitor KPS during the follow-up management of patients. Further studies with larger sample sizes and more detailed follow-up data are needed to investigate the prognostic factors that could guide us in the daily management and clinical practice of patients with lung cancer after screening.

## Supplementary Information

Supplementary Material 1.

## Data Availability

The datasets used during the current study are available from the corresponding author on reasonable request.
