# Supplementary material for: Prognostic factors of survival in patients with lung cancer after low-dose computed tomography screening: a multivariate analysis of a lung cancer screening cohort in China
Source: BMC Cancer. 2025 Apr 9;25:646. doi: 10.1186/s12885-025-14036-9 (PMC11984240; doi:10.1186/s12885-025-14036-9)
Supplement: Supplementary file 1 — Supplementary Material 1. [file 12885_2025_14036_MOESM1_ESM.docx]

**Health Questionnaire of Lung Cancer for Residents Aged 40 and Above in Minhang District, Shanghai**

Questionnaire Code:□□□□□

Part 1 General Information

101. Name: ___________________
102. Gender: ______
103. Phone number: _______________
104. Date of birth: Year _____ Month _____
105. Place of birth: _______________________
106. Household registration address: ________________________________
107. Current residential address: ________________________________
108. Education level:
1) Primary school or below 2) Middle school 3) High school

4) College 5) Bachelor degree or above

109. Marital status:
1) Single 2) Married 3) Divorced 4) Widow 5)Others

110. What is your personal monthly income (CNY) in the past 3 months:
1) <1000 2) 1000−2000 3) 2000−3999

4) 4000−5999 5) 6000−9999 6) ≥10,000

Part 2 Pulmonary Disease

201. Have you ever smoked? 1)Yes 2)No

202. Do you have a previous medical history of pulmonary diseases?

1. Tuberculosis: 1) Yes 2) No

2. Chronic obstructive pulmonary disease: 1) Yes 2) No

Thank you for your cooperation!

Interviewer’s Signature: ____________________
Date: Year ______ Month ______ Day ______

Made by Shanghai Minhang Center for Disease Control and Prevention
